# A bibliometric analysis of global research hotspots and development trends in diabetic wound treatment

**DOI:** 10.3389/fcdhc.2025.1603206

**Published:** 2025-06-20

**Authors:** Yin Wen, Kaiyu Nie

**Affiliations:** ^1^ Department of Burns and Plastic Surgery, Affiliated Hospital of Zunyi Medical University, Zunyi, China; ^2^ The 2011 Collaborative Innovation Center of Tissue Damage Repair and Regeneration Medicine, Affiliated Hospital of Zunyi Medical University, Zunyi, China; ^3^ The Collaborative Innovation Center of Tissue Damage Repair and Regeneration Medicine, Zunyi Medical University, Zunyi, China

**Keywords:** bibliometric, diabetic wounds, treatment, prognosis, management

## Abstract

**Background:**

Diabetic wounds are a serious complication for diabetic patients, characterized by refractoriness, high recurrence rates, and susceptibility to infection. Although current guidelines recommend evidence-based treatment strategies, clinical outcomes remain suboptimal. This paper reviews the current research status and development trends in diabetic wound treatment.

**Methods:**

Articles on diabetic wound treatment published between 2014 and 2023 were identified using the Web of Science Core Collection database, resulting in a total of 9,099 articles. Bibliometric methods were employed to analyze authors, institutions, countries, journals, keywords and references using CiteSpace and VOSviewer.

**Results:**

China has published the most articles in the field, followed by the United States. Shanghai Jiao Tong University is the leading institution in diabetic wound treatment research, and David G. Armstrong from the United States has made significant contributions to this field. “Wound Repair and Regeneration” was identified as the most influential journal. Cluster analysis of keywords revealed four main categories: (1) mechanisms of diabetic wound healing, (2) prognosis, (3) treatment, and (4) management.

**Conclusion:**

This paper systematically reviews the research on diabetic wound treatment from 2014 to 2023, outlining and forecasting global research hotspots and trends. Future research is expected to focus on treatment strategies for diabetic wounds, while interdisciplinary collaboration and advancements in intelligent management technologies have the potential to improve patient outcomes.

## 1 Introduction

With the ongoing development of the global economy, the number of people with diabetes is steadily increasing. Currently, over 500 million people worldwide are affected by diabetes, and this number is projected to exceed 750 million by 2045 (1). Diabetic wounds are a severe complication associated with diabetes, with diabetic foot ulcers being among the most critical. Data indicate that between 7.2%-15% of diabetic patients will develop diabetic foot ulcers. Of those, approximately 7.7%-44% will experience recurrence of the ulcers after they have healed. Furthermore, 50%-60% of these ulcers are prone to secondary infections and, in severe cases, may necessitate amputation. This situation significantly increases both the personal burden on patients and their risk of mortality (2).

The updated International Working Group on the Diabetic Foot (IWGDF) guidelines outline several treatment strategies for diabetic wounds, particularly diabetic foot ulcers. These strategies include antimicrobial treatments, necrotic tissue debridement, enhancement of tissue perfusion, wound dressing, negative pressure therapy, oxygen therapy, pressure offloading, nutritional support, and psychological counseling (3, 4). Despite these comprehensive approaches, treatment outcomes for diabetic wounds remain unsatisfactory. The underlying reasons for this lack of success are unclear—whether they stem from patient compliance issues or variations in medical practices. In an era marked by advanced science and technology, finding effective ways to manage diabetic wounds and alleviate the burden on patients and society is a critical and ongoing challenge.

In this study, we investigate the century-old challenge of treating diabetic wounds by examining research conducted over the past decade. Our goal is to summarize the current trends in diabetic wound treatment research and to analyze and forecast future research hotspots.

## 2 Materials and methods

### 2.1 Database and search strategy

**Figure 1:**
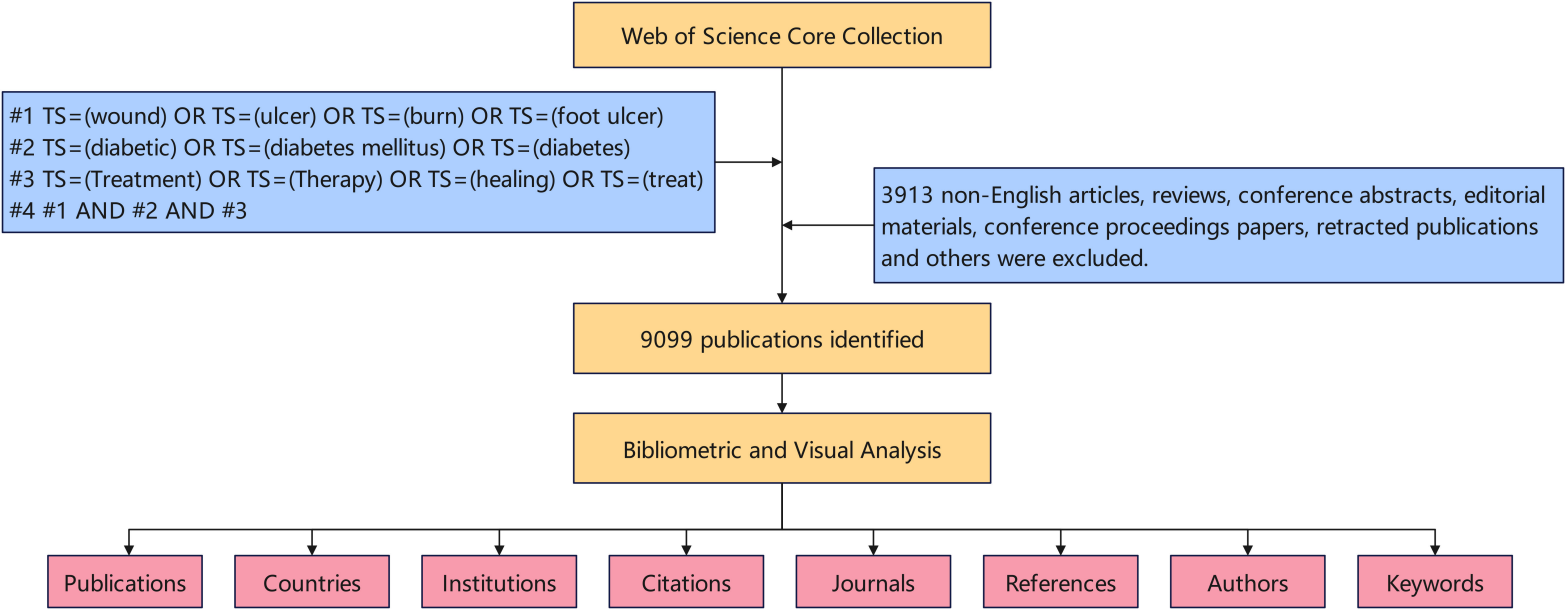
Detailed flowchart steps of the search strategy in screening publications.

We conducted a search by TS (Topic) in the Web of Science Core Collection (WOSCC) and collected all the publications of this study, which included more than 12,000 highly influential high-quality scientific journals. **Figure 1** provides a detailed overview of the data retrieval procedures and inclusion criteria employed in this research. The search strategies are also outlined in **Figure 1**. Only English-language articles published between January 1, 2014, and December 31, 2023.

### 2.2 Data analysis

The authors, title, publication year, institution, country/region, keywords, citations, abstracts, and references were obtained from the WOSCC database in plain text format. To calculate the impact factor, Journal Citation Reports (JCR) 2023 was utilized. Data qualifying for inclusion in this study were extracted using the VOSviewer 1.6.19 program and subsequently imported into CiteSpace 6.2.R6 and Microsoft Excel 2021. VOSviewer, a traditional bibliometric analysis tool, was employed for visual analysis in bibliometric research (5). CiteSpace was used to analyze co-occurrence and centrality within collaborative networks among countries, authors, and institutions (6). Detailed usage instructions for VOSviewer are available at https://www.vosviewer.com/getting-started, while CiteSpace can be accessed and downloaded from https://citespace.podia.com/, which offers text and video tutorials. Differences in publication volume between countries were presented using https://amcharts.com/editor/map/.

## 3 Results

### 3.1 Analysis of the number of publications and citations

**Figure 2:**
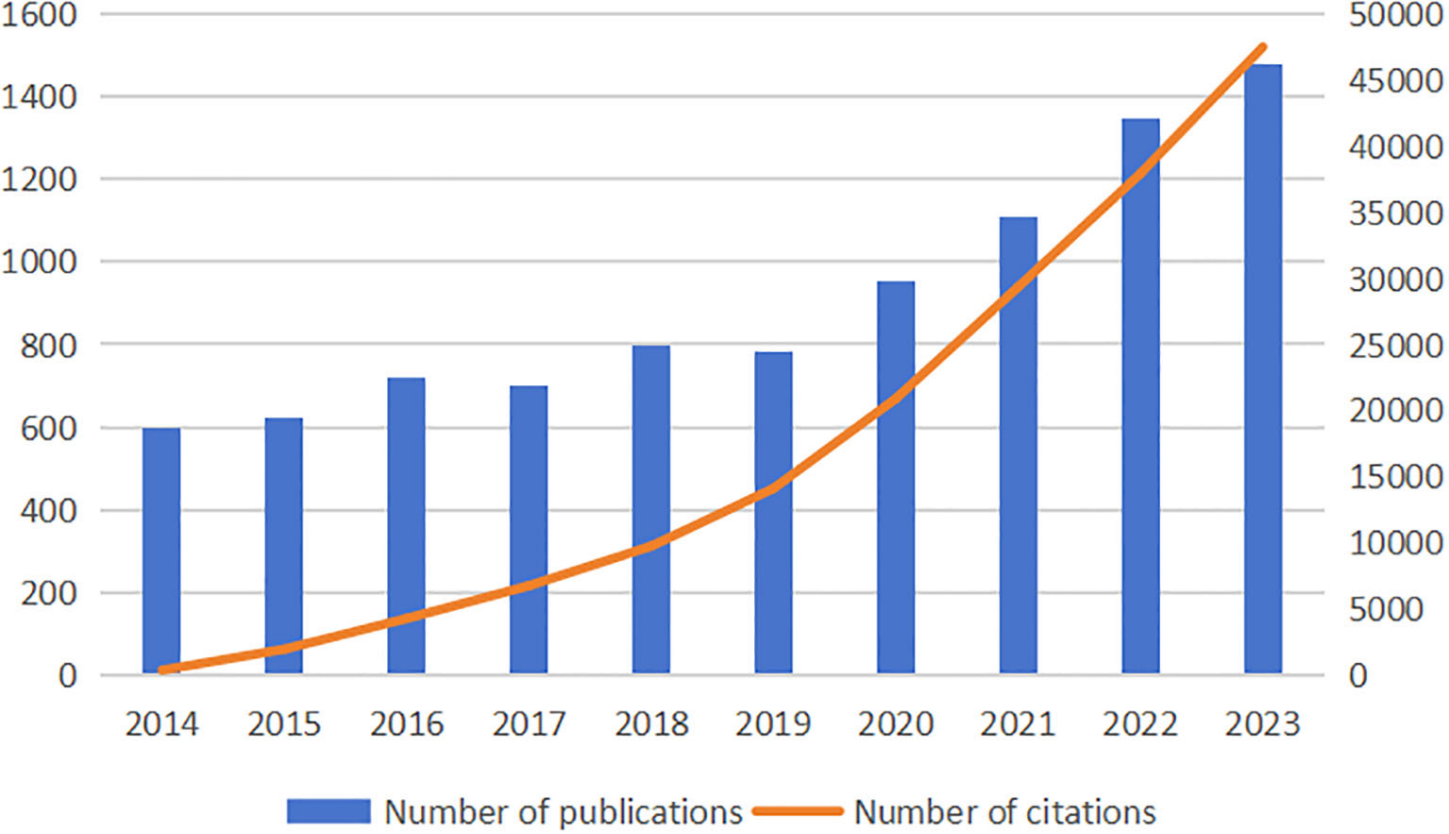
The annual number of publications and citations on treatment of diabetes wound from 2014 to 2023.

From 2014 to 2023, there were 9099 papers on treatment of diabetes wound, according to comprehensive manual screening. The numbers of publications and citations are shown in **Figure 2**. We observed that the number of papers published and citations continued to increase from the year 2014 onward.

### 3.2 Analysis of contributions of prolific authors and co-cited authors

As shown in **Table 1**, among the top 10 most prolific authors in this discipline, only two authors are from China, while the majority are from the United States. Furthermore, most of the top 10 co-cited authors originate from the USA, with the remaining few from the UK and the Netherlands.

**Table 1:** Top 10 prolific authors and co-cited authors.

Rank	Author	Country	Documents	Citation	Average article citation	Co-cited author	Country	Citation
1	David G Armstrong	USA	61	2857	46.84	David G Armstrong	USA	1755
2	Lawrence A Lavery	USA	38	1668	43.89	Benjamin A Lipsky	UK	1172
3	Wang wei	China	37	1383	37.38	Lawrence A Lavery	USA	955
4	Dennis P Orgill	USA	34	866	25.47	Vincent Falanga	USA	844
5	Li yan	China	33	1058	32.06	Andrew JM Boulton	UK	832
6	Mohammad Bayat	USA	30	684	22.80	Harold Brem	USA	633
7	Robert S Kirsner	USA	29	1079	37.21	William J Jeffcoate	UK	561
8	Peter A Lazzarini	Australia	28	722	25.79	Sicco A Bus	Netherlands	560
9	Christopher E Attinger	USA	28	286	10.21	David J Margolis	USA	548
10	Jaap J Van Netten	Netherlands	27	1244	46.07	Chandan K Sen	USA	538

**Figure 3:**
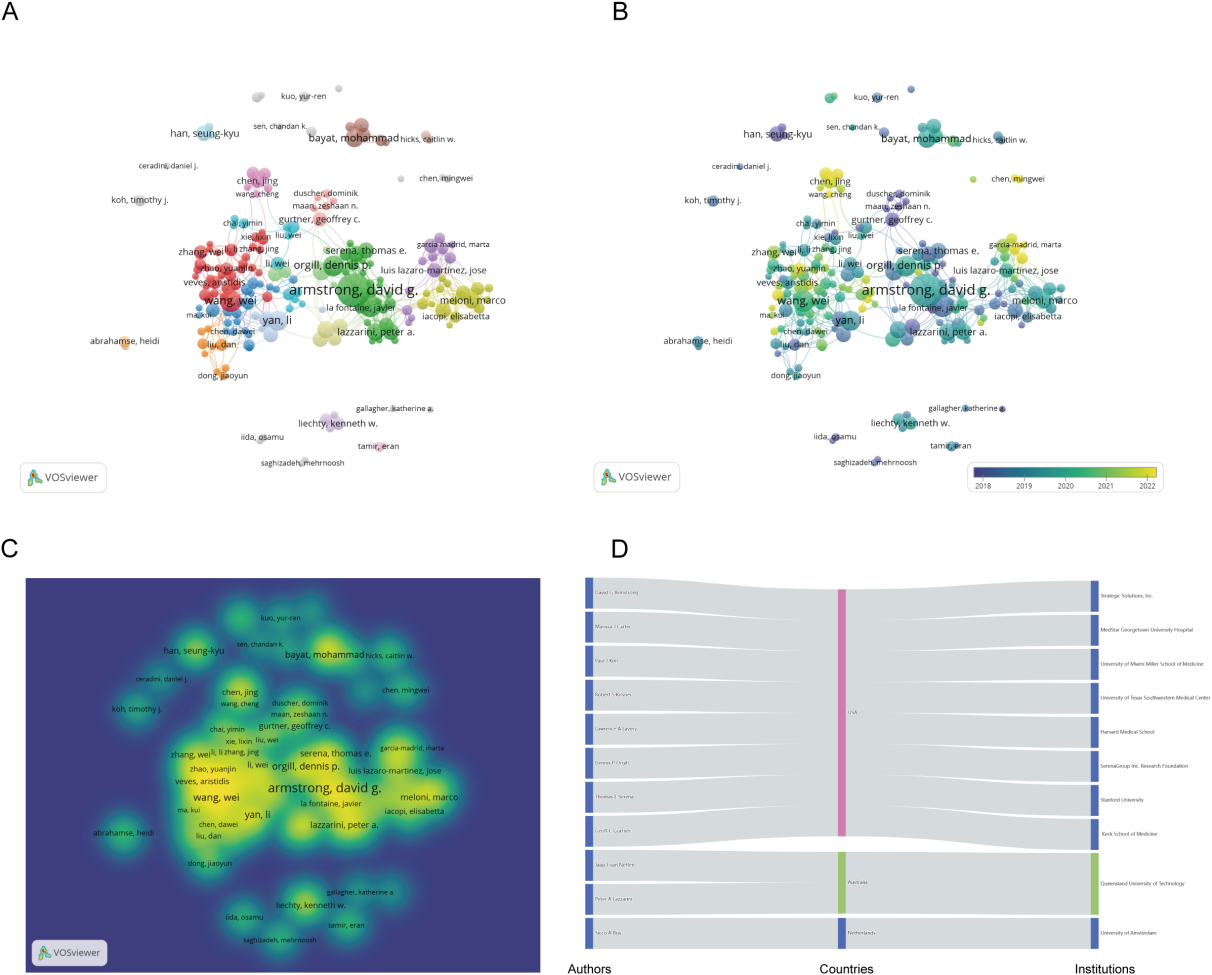
Co-authorship analysis of the excellent authors in the field of diabetes wound. **(A)** A network visualization map. **(B)** An overlay visualization map. **(C)** A density visualization map. **(D)** Countries and institutions of these distinguished authors.

**Figure 3** presents a collaborative network of authors with more than 10 publications. The network visualization map (**Figure 3A**) displays 31 clusters. David G Armstrong stands out as the most connected author, with a total of 79 links. The overlay map indicates the formation of new research teams led by Chinese scholars Chen Jiang and Wang Cheng (**Figure 3B**). The density visualization map illustrates that Chinese researchers have been notably active in diabetic wound research in recent years (**Figure 3C**). Finally, **Figure 3D** depicts the relationships among the top 11 authors, including their countries and affiliated institutions.

### 3.3 Analysis of the contributions of journals

**Figure 4:**
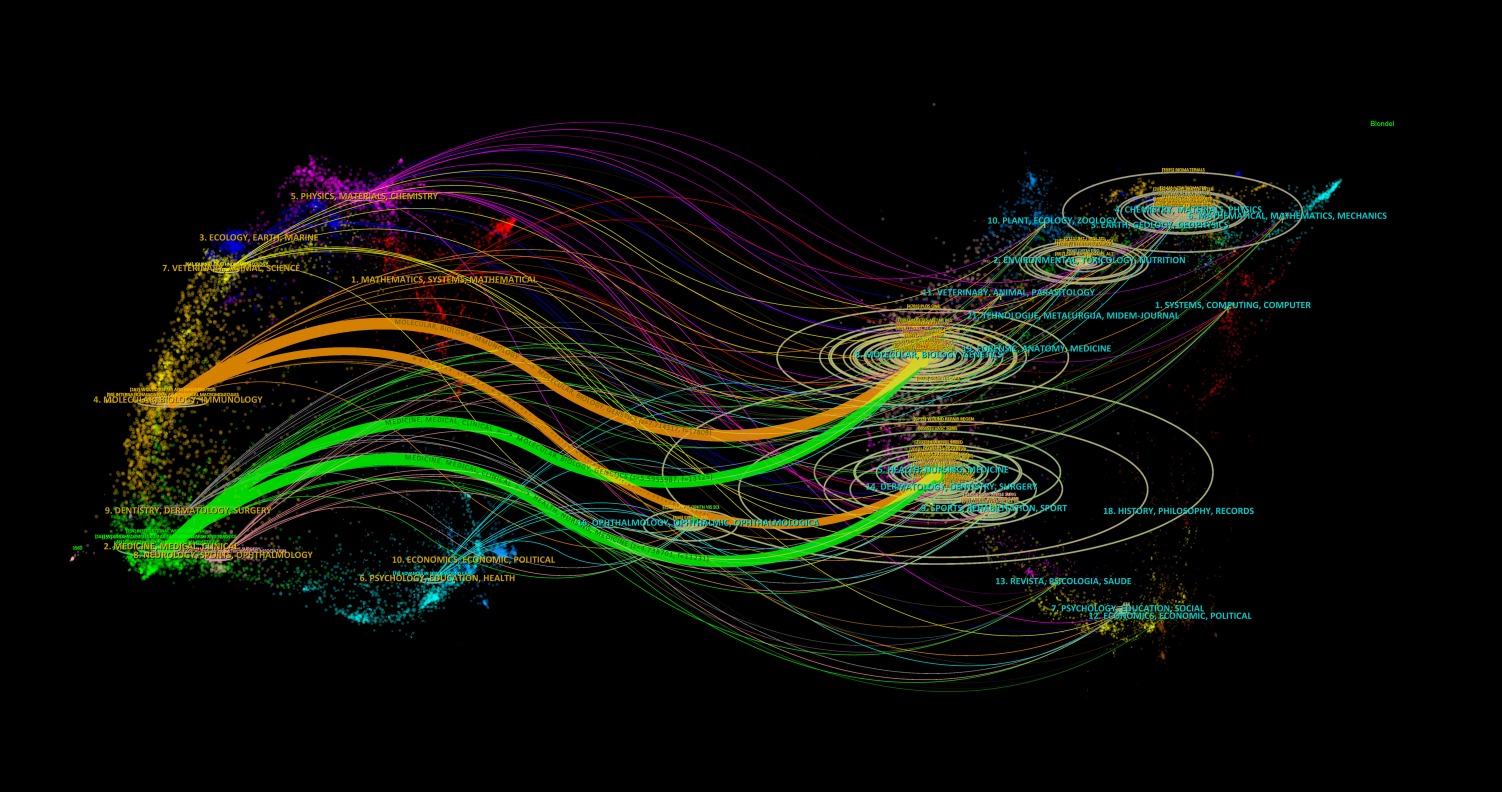
The dual-map overlay of journals related to treatment of diabetes wound.

To illustrate the distribution of citing and cited journals, we used a dual-map overlay atlas. In **Figure 4**, the geographic area on the left represents the citing journals, while the map on the right represents the cited journals. Different disciplines are indicated by varying line colors. The citing journals primarily focus on molecular biology, immunology, genetics, nursing, surgery, medicine, clinical studies, and chemistry. In contrast, the cited journals mainly cover chemistry, environmental science, genetics, molecular biology, medicine, health, nursing, biology, dermatology, surgery, nutrition, and toxicology.

**Figure 5:**
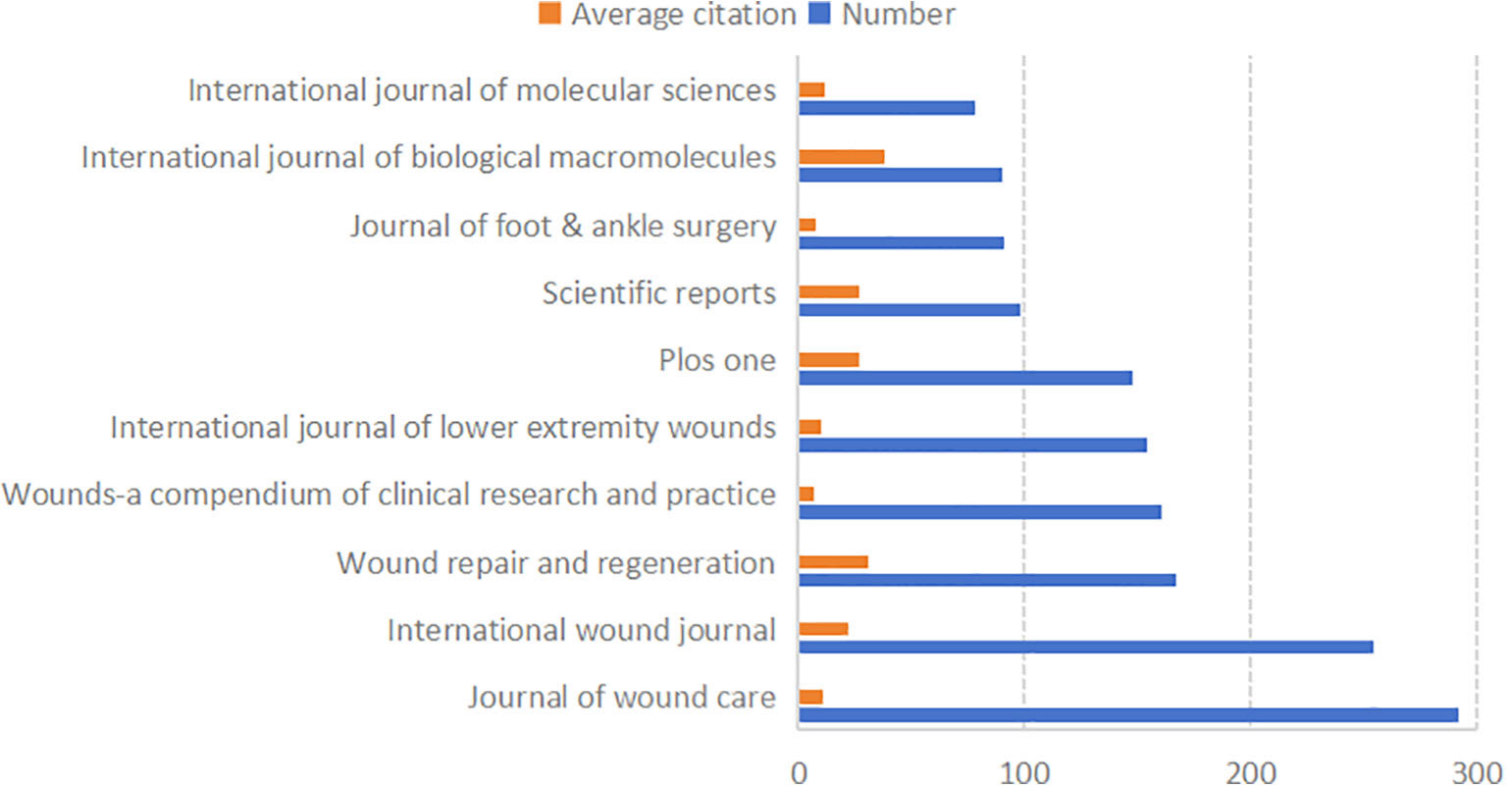
The average citation and total publication number of the top 10 journals.

A total of 1,729 journals have published articles in this field. **Figure 5** lists the 10 most prolific journals, along with their average citation rates and total publication numbers in this domain. Most of these top 10 journals are classified as JCR Q1 and Q3.

### 3.4 Analysis of the contributions by institutions

**Figure 6:**
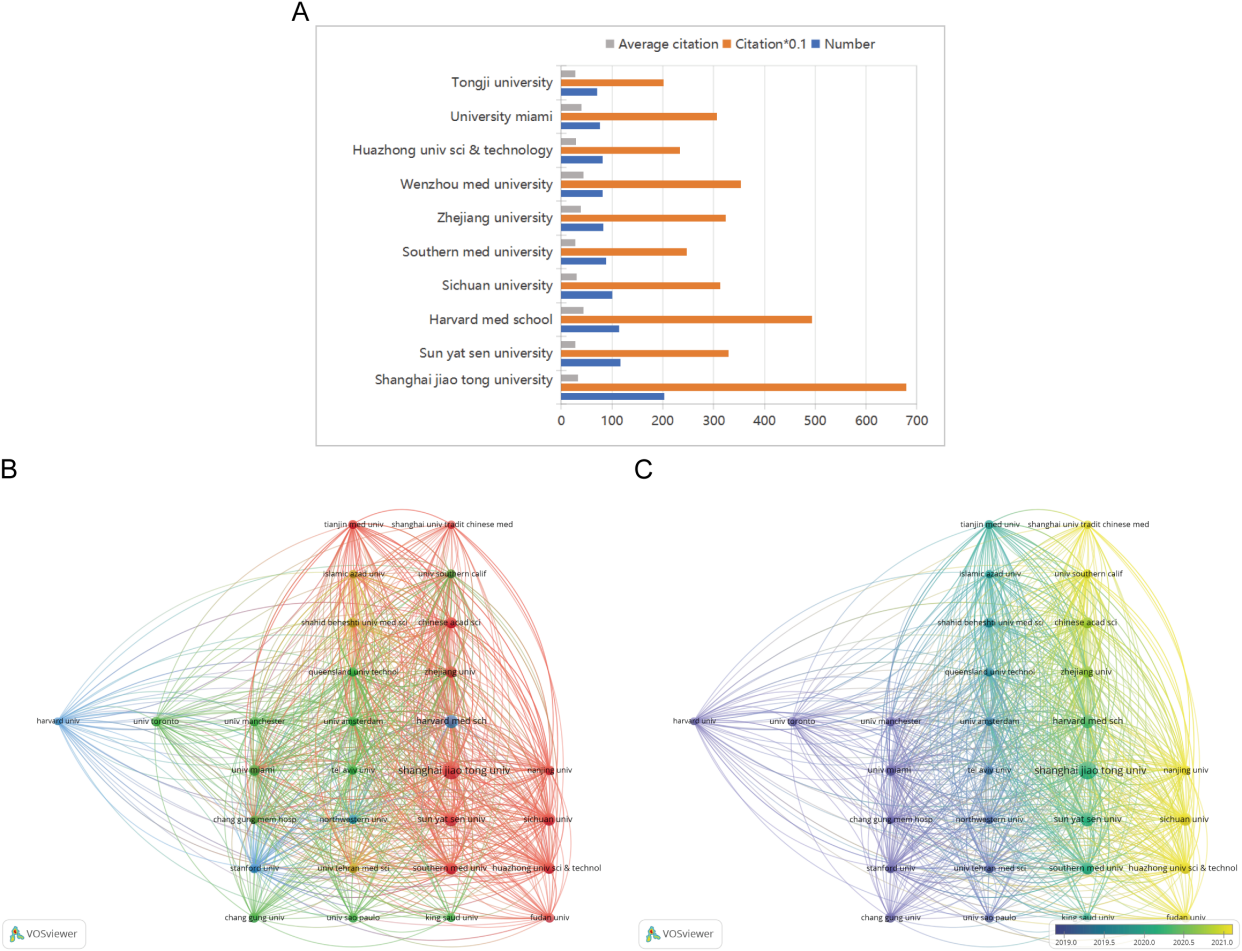
**(A)** The total number of publications, the average number of citations and total number of citations of the top 10 institutions. **(B)** A network visualization map. **(C)** An overlay visualization map.

**Figure 6A** illustrates the top 10 most productive institutions, with eight of them located in China. The analysis focused on institutions with at least 40 publications, using VOSviewer to identify 29 institutions involved in co-authorship. **Figure 6B** depicts the institutional co-authorship network, which includes 29 institutions and is organized into 5 clusters. Recent trends indicate that Chinese institutions are increasingly influential in the field of diabetic wound research, as demonstrated by the overlay map in **Figure 6C**, which shows the historical trend of article production.

### 3.5 Analysis of the contributions of countries

**Figure 7:**
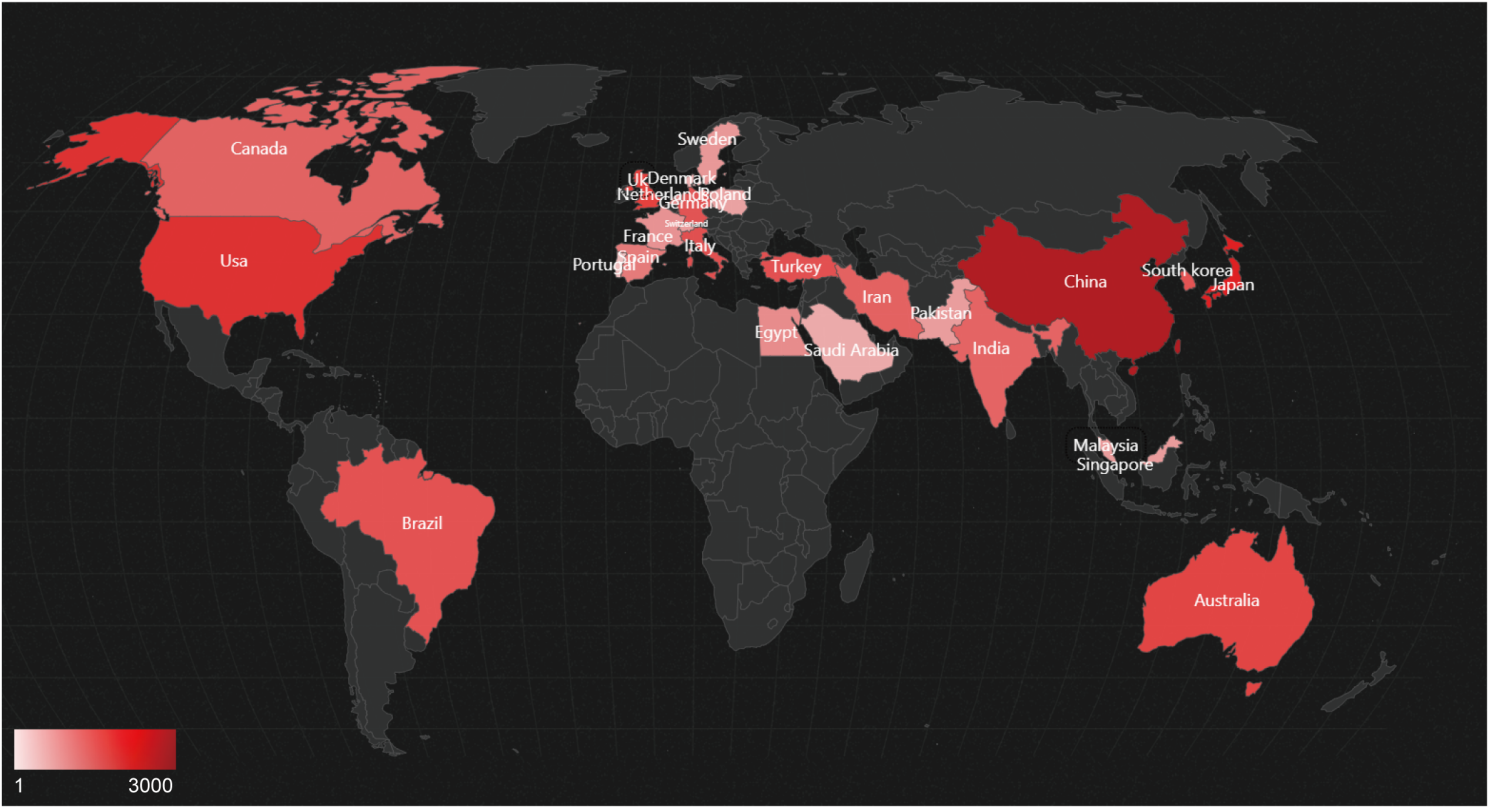
Geographic distribution of global publications on treatment of diabetes wound.

**Figure 8:**
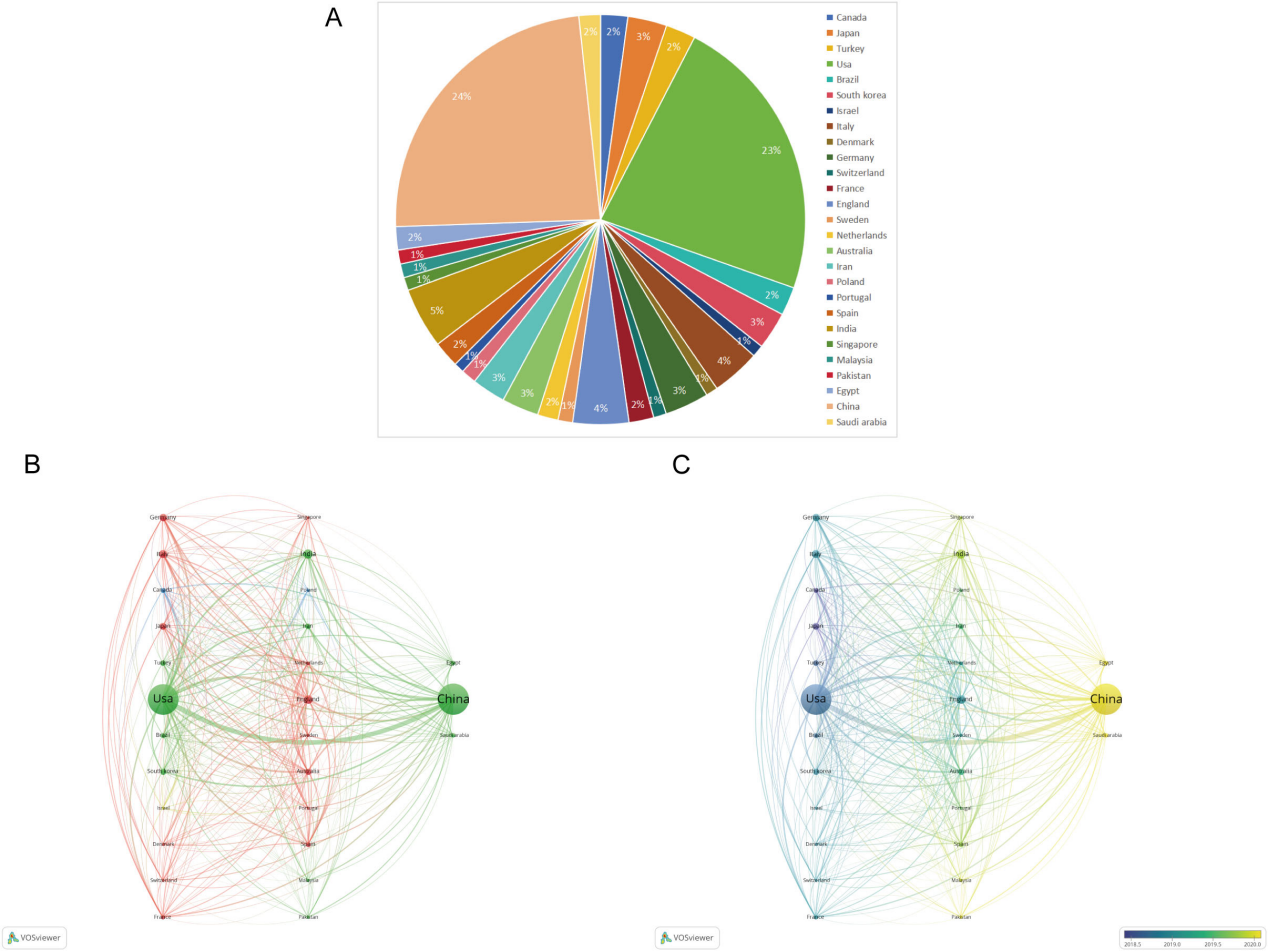
**(A)** Pie chart of the 27 productive countries/regions. **(B)** Network visualization map of the top 27 countries’ collaboration. **(C)** An overlay visualization map.

**Figure 7** displays the geographical distribution of global publications in the field of diabetic wounds across 27 countries and regions. **Figure 8A** provides a comparison of publication proportions among these countries. **Figure 8B** examines the co-authorship network between these 27 countries using VOSviewer. As shown in overlay visualization map (**Figure 8C**), China has recently actively involved in substantial research collaborations.

### 3.6 Analysis of a highly cited study

**Figure 9:**
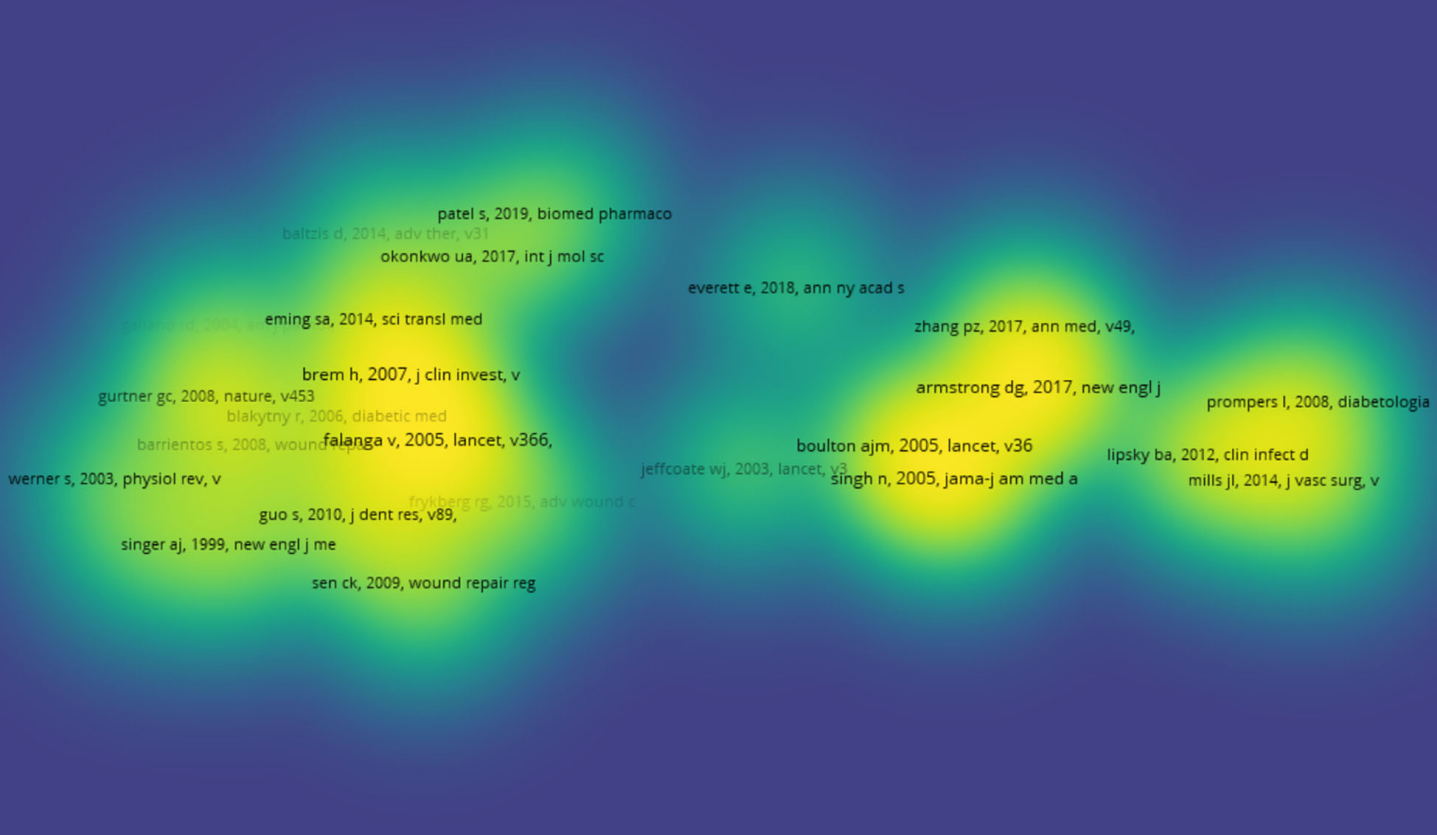
Density visualization map of the top 27 co-cited references.

The top 10 articles with the highest citation frequencies are listed in **Table 2**. The article titled ‘Diabetic Foot Ulcers and Their Recurrence’ published in the New England Journal of Medicine in 2017 was cited the most. Out of 232823 references 27, articles were cited more than 150 times are shown in **Figure 9**.

**Table 2:** The top 10 co-citation references related to treatment of diabetes wound.

Rank	Year	First author	Title	Source	Citation
1	2017	Armstrong DG	Diabetic Foot Ulcers and Their Recurrence	New England Journal of Medicine	644
2	2005	Falanga V	Wound healing and its impairment in the diabetic foot	Lancet	606
3	2005	Singh N	Preventing foot ulcers in patients with diabetes	JAMA	503
4	2005	Boulton AJ	The global burden of diabetic foot disease	Lancet	428
5	2007	Brem H	Cellular and molecular basis of wound healing in diabetes	Journal of Clinical Investigation	425
6	2012	Lipsky BA	Executive summary: 2012 Infectious Diseases Society of America clinical practice guideline for the diagnosis and treatment of diabetic foot infections	Clinical Infectious Diseases	355
7	2010	Guo S	Factors affecting wound healing	Journal of Dental Research	326
8	2008	Gurtner GC	Wound repair and regeneration	Nature	311
9	2014	Eming SA	Wound repair and regeneration: mechanisms, signaling, and translation	Science Translational Medicine	278
10	2009	Sen CK	Human skin wounds: a major and snowballing threat to public health and the economy	Wound Repair and Regeneration	274

### 3.7 Analysis of keywords

**Figure 10:**
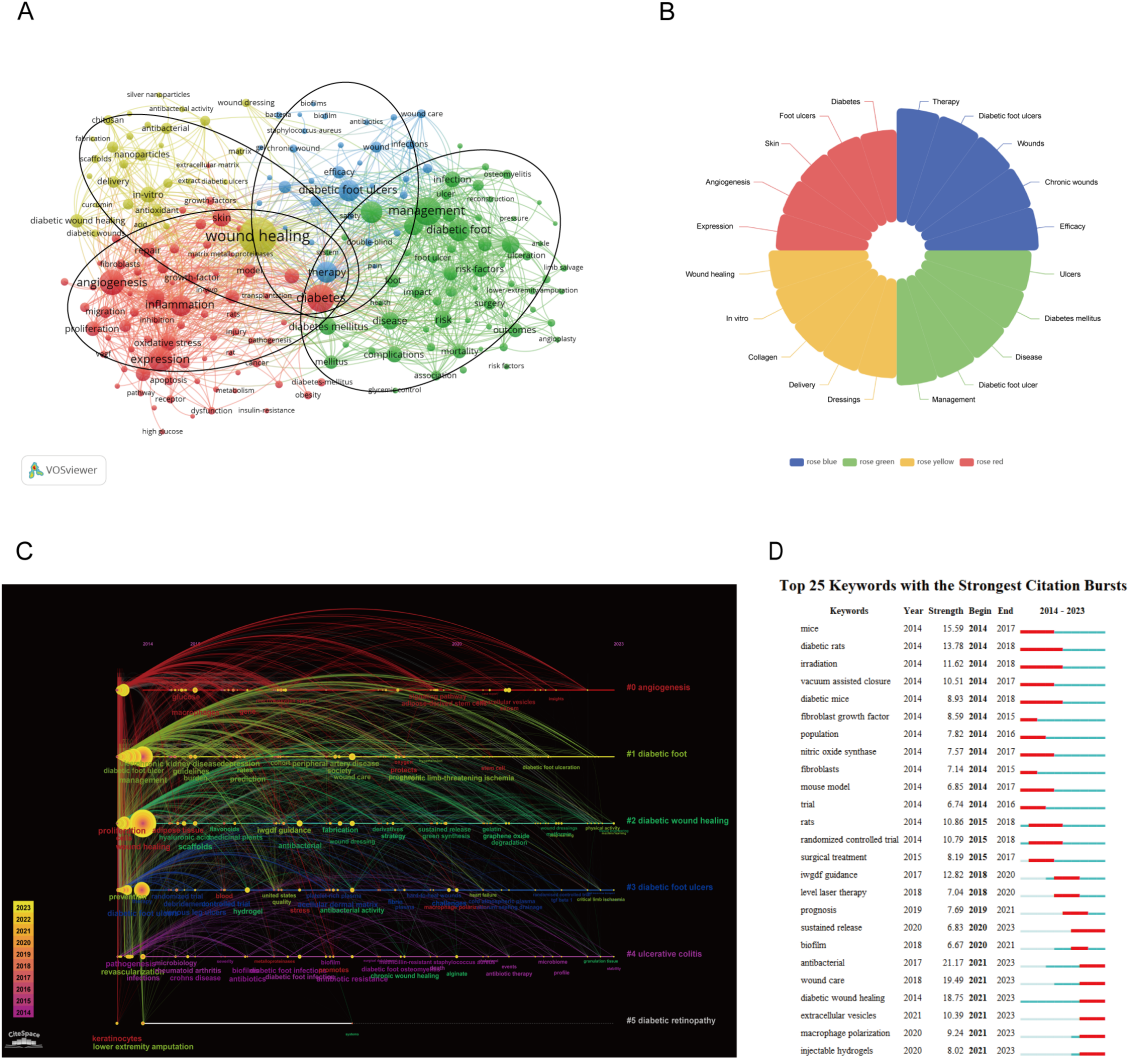
**(A)** A network visualization map. **(B)** Keywords that are particularly important in the four groups. **(C)** A timeline and keyword clustering display for the diabetics wound. **(D)** The top 25 terms with the most significant diabetics wound citation bursts.

**Figure 10A** shows keywords with a frequency of higher than 58. In total, 200 high-frequency keywords were selected from 9099 studies, and divided into four clusters. Cluster 1 (red cluster): mechanism of diabetic wound, including gene expression, cell migration and proliferation, oxidative stress, growth-factor, inflammation, and angiogenesis; cluster 2 (blue cluster): prognosis, including efficacy, safety, complications; cluster 3 (yellow cluster): treatment, include antioxidant, scaffolds, antimicrobial, silver nanoparticles, delivery, curcumin; cluster 4 (green cluster): management, include pain, risk factors, diabetes mellitus, pressure, health, glycemic control, prevention of infection. As shown in **Figure 10B**, the top five keywords with the highest occurrences across the four major clusters. The timeline picture can show the period and time trend of different research keywords. According to **Figure 10C**, angiogenesis, diabetic foot, and diabetic wound healing are the longest-lasting hotspots, which lasted from 2014 to the present. Burst word analysis can reveal hot research in a research field during a specific period. We show the top 25 keywords with the strongest citation bursts in **Figure 10D**.

## 4 Discussion

### 4.1 Global research status and trends

According to an analysis of national publication data, China and the United States have made substantial contributions, with a total of 4,688 articles, accounting for 51.52% of the overall literature. Additionally, data on author collaboration strength indicate that the top three authors are from China and the United States, suggesting a closely connected and influential group of researchers from these countries. From the perspective of journal publications and citations, “Wound Repair and Regeneration” stands out as a significant journal in the field of diabetic wound research, offering highly valuable and relevant papers.

In our analysis of the top 10 institutions, we found that eight are based in China. The top three institutions are Shanghai Jiao Tong University, Sun Yat-sen University, and Harvard Medical School.

China and the United States have significantly contributed to the literature on diabetic wounds, with their leading positions attributable to national strategic orientations and disciplinary integration models. China benefits from strong policy-driven initiatives and collaborative disciplines, whereas the United States primarily emphasizes research innovation mechanisms and the connections among industry, academia, and research institutions. This will further promote the output of their research results.

### 4.2 Research keyword analysis

Through cluster analysis of 200 keywords with frequencies exceeding 58, these keywords were categorized into four main clusters: Cluster 1 (red cluster) focuses on the mechanism of diabetic wounds healing; Cluster 2 (blue cluster) covers prognosis; Cluster 3 (yellow cluster) relates to treatment; and Cluster 4 (green cluster) pertains to management. These keywords highlight the primary research directions and topics of interest within the co-occurrence analysis.

By examining the timeline of keywords and the top 20 emerging terms, a high frequency of keywords related to mechanisms and treatments from 2014-2023 indicates that these areas have been research focal points. The recent emergence of terms such as “antimicrobial,” “wound care,” “diabetic wound healing,” “extracellular vesicles,” “macrophage polarization,” and “injectable hydrogels” suggests that these are recent research hotspots and may also be future areas of interest. Furthermore, studies related to management have also gained prominence, often intertwining with prognosis, in line with the results of our cluster analysis. By analyzing these four categories, we can explore the current research hotspots and directions in the field of diabetic wound therapy.

### 4.3 Red cluster: mechanism of diabetic wounds healing

The complexity of wound healing arises from the intricate coordination between various tissues and cells, involving processes such as cell migration, proliferation, matrix deposition and remodeling, inflammatory response, and angiogenesis (7). However, these processes are often significantly disrupted in diabetic patients due to the effects of prolonged hyperglycemia, leading to impaired wound healing (8). Recent studies have highlighted the impact of aberrant cell signaling pathways on wound healing under high glucose conditions, particularly emphasizing the role of dysregulated macrophage polarization and impaired angiogenesis (9, 10).

Macrophages are crucial immune cells that regulate the inflammatory process and can be activated and polarized into different phenotypes. M1 macrophages exhibit pro-inflammatory properties, while M2 macrophages possess anti-inflammatory capabilities that aid in wound healing by promoting vascular regeneration, cell proliferation, and inhibiting inflammation. During the inflammatory phase of diabetic wounds, macrophages release various inflammatory cytokines such as tumor necrosis factor (TNF)-α and interleukin (IL)-6, which impede the progression of the healing process into the proliferative phase (7). Moreover, sustained activation of the NF-κB signaling pathway in high glucose conditions triggers a chronic inflammatory response, further delaying the wound healing process (11).

Impaired angiogenesis is a significant factor contributing to delayed wound healing in diabetes. Vascular endothelial cells (ECs) are crucial for forming new blood vessels, but their growth and function are often compromised, leading to difficulties in vessel formation and wound repair (12). In hyperglycemic conditions, endothelial cell damage is caused by several factors. Chronic high glucose levels result in direct damage to endothelial cells through the formation of advanced glycation end products (AGEs). AGEs modify cellular proteins and DNA via non-enzymatic glycosylation, disrupting normal cellular functions (13, 14). Additionally, hyperglycemia induces mitochondrial dysfunction, leading to the production of excessive reactive oxygen species (ROS). This damages the mitochondrial membrane potential and further impairs wound healing (15).

Oxidative stress from elevated free radicals and oxidative substances exacerbates endothelial cell damage, activates inflammatory pathways, and intensifies the inflammatory response. The chronic low-grade inflammation in diabetes promotes the release of inflammatory mediators such as IL-6 and TNF-α, which not only worsen endothelial cell damage but also increase blood vessel permeability and impede angiogenesis (16, 17). Furthermore, reduced angiogenesis in a hyperglycemic environment affects endothelial cell proliferation and migration, leading to insufficient new vessel formation, inadequate nutrient supply, and a hypoxic environment. This, in turn, triggers and worsens inflammation, further exacerbating endothelial cell damage (18, 19).

### 4.4 Blue cluster: prognosis

Diabetic foot ulcers and amputations significantly increase the risk of mortality (2). Research indicates that the 5-year mortality rate for individuals with a diabetic foot ulcer is approximately 30%, exceeding 70% for those with a major amputation (2). Consequently, researchers are focusing on early identification and intervention for diabetic wounds. A cohort study showed that by using statistics and machine learning techniques on patient data, it was demonstrated that genetic information has predictive potential in diabetic wound healing. This data includes clinical factors, circulating endothelial precursor cells (CEPCs), and fine predictive sequencing of the NOS1AP gene (20). Additionally, in patients with diabetic peripheral neuropathy, enhanced sensory testing using a tuning fork has shown that vibration sensitivity of the distal phalanx is highly accurate and clinically relevant for predicting the development of calluses and skin ulcers (21).

In addition to research on early prediction, many studies have concentrated on effectively assessing prognosis and improving long-term survival in patients with diabetic wounds (2). For instance, Jia et al. investigated the granulation tissue in negative pressure wound therapy (NPWT) using label-free quantitative mass spectrometry. Their analysis revealed that NPWT significantly altered protein expression in granulation tissue, particularly in processes related to detoxification, antioxidant activity, inflammatory regulation, and extracellular matrix modification. Notably, changes in proteins such as PRDX2, PROS1, CTSS, and ITIH4 offer valuable insights into potential biomarkers for assessing responses to diabetic wound treatments in the future (22). Bender et al. examined characteristics of diabetic wounds, including necrosis, wound size, and patient age, to develop a simple predictive model based on bedside wound characteristics. This model effectively predicts the healing outcome of diabetic foot ulcers, whether the wound size is likely to increase or decrease (23). Artificial intelligence, as a cutting-edge technology, holds significant promise for integration into diabetic wound treatment (24). Pereira MG et al. employed machine learning and decision tree algorithms to evaluate the risk of ulcer healing in diabetic feet. They found that factors such as disease perception, ulcer duration, and biochemical markers (e.g., IL-6, microRNA-146a-5p, PECAM-1, and angiopoietin-2) had substantial predictive value for healing outcomes (25). The high recurrence rate of diabetic foot ulcers post-healing is another critical issue. To address this, Wang et al. developed and validated three prediction models to assess the risk of ulcer recurrence. Model 1 uses social and clinical indicators; Model 2 adds factors such as medication, blood pressure, and body mass index; and Model 3 further incorporates laboratory indicators. Model 3 demonstrated the highest performance in predicting the risk of diabetic foot ulcer recurrence, with an AUC value of 0.899 and the greatest sensitivity and specificity. While Models 1 and 2 are effective for general screening, Model 3 provides a more accurate screening tool (26).

### 4.5 Yellow cluster: treatment

The treatment strategies recommended by the IWGDF guidelines for diabetic wound care include antimicrobial therapy, necrotic tissue resection, enhanced tissue perfusion, wound dressing, negative pressure therapy, oxygen therapy, and decompression (3, 4). Although these evidence-based approaches have proven effective, clinical management of diabetic wounds often remains suboptimal. The bacteria causing wound infections in diabetes are mainly Gram-positive and Gram-negative. Among them, the most common ones are Staphylococcus aureus, Escherichia coli, Pseudomonas aeruginosa, Klebsiella pneumoniae. In fungal infections, Candida albicans is also relatively common, especially in patients with low immunity. Furthermore, the prevalence rate of multiple microbial infections is 22.8% (27, 28). In addition to using conventional antibiotics, researchers are exploring novel treatments such as stem cells, extracellular vesicles, miRNAs, nanoenzymes, hydrogels, and microneedles (28–32).

Stem cells can accelerate diabetic wound healing by promoting cell regeneration, regulating the microenvironment, and reducing inflammation (33). However, their application is constrained by ethical issues and potential immunogenicity. Recent studies have highlighted extracellular vesicles, such as stem cell-derived exosomes, as superior therapeutic carriers compared to synthetic nanoparticles. These vesicles offer extended circulatory half-life, low immunogenicity, biocompatibility, ease of modification, and stable payload (34). Additionally, the miRNAs contained in exosomes have garnered significant attention. miRNAs are non-coding RNAs that regulate protein expression and associated pathways post-transcriptionally, and they have been extensively studied in diabetic wound management (35). Notably, encapsulating therapeutic miRNAs in exosomes protects them from ribonuclease degradation, increases their concentration in wound tissue or cells, and enhances wound healing. Tao et al (36). utilized gene overexpression technology to generate exosomes overexpressing miR-126-3p from synovial mesenchymal stem cells (SMSCs), demonstrating accelerated wound reepithelialization, angiogenesis, and collagen maturation in diabetic mice. Similarly, Yan et al (37) used miR-31-5p loaded in ex vivo milk. However, the clinical application of engineered exosomes faces challenges, including stability and persistence in wounds. Nanoenzymes also encounter similar issues (38).

Hydrogels and microneedles are innovative biomaterials playing a crucial role in the treatment of diabetic wounds. Hydrogels create a moist healing environment, promote wound repair, reduce infection risks, and facilitate drug or growth factor release to accelerate healing (10). On the other hand, microneedles can painlessly penetrate the skin, directly delivering drugs or therapeutic agents to deeper tissues, thereby enhancing the therapeutic effect and minimizing the dosage of drugs as well as the pain and discomfort associated with traditional injections. They can also inhibit pathogenic bacteria such as Escherichia coli and Staphylococcus aureus, exerting an antibacterial effect and promoting the healing of diabetic wounds (39, 40). Recent research indicates that microneedles made from hydrogels offer these combined advantages (41). Furthermore, the use of hydrogels or microneedles has been shown to significantly improve diabetic wound healing (10, 41).

Given the persistent and multifactorial nature of diabetic wound treatment, researchers have addressed the limitations of other therapeutic agents—such as exosomes, miRNA, or nanoenzymes—by integrating them into multifunctional hydrogels or microneedles, they not only exhibit sustained release properties but also possess antimicrobial, anti-inflammatory, antioxidant, and angiogenic effects (38, 39, 42, 43). Moreover, recent studies have combined phototherapy and electrotherapy with these hydrogels or microneedles, providing multiple options for personalized and precise diabetic wound treatment (30, 44, 45). These combined therapies using combined materials may become breakthrough points and provide more research directions for the treatment of diabetic wounds. It is also noteworthy that the application of AI in diabetic wound care can enable real-time dynamic monitoring, representing a promising strategy for future advancements (46, 47).

### 4.6 Green cluster: management

The management of diabetic wounds is undergoing significant transformation, with innovations in management approaches standing out. Remote monitoring technology and intelligent management platforms now enable medical teams to track patients’ wound conditions and blood glucose levels in real time. This capability allows for personalized treatment recommendations and ensures centralized management and intelligent data analysis (47, 48). Additionally, interdisciplinary collaboration has become crucial in enhancing treatment outcomes. Experts in endocrinology, surgery, nursing, and nutrition work together to provide comprehensive care through blood glucose control, specialized wound management, meticulous care, and nutritional support (49–51). This integrated approach not only accelerates wound healing but also optimizes the overall health of patients and significantly improves their quality of life.

### 4.7 Limitations

Our bibliometric study has several limitations. Firstly, it only covers literature published in English, leaving out significant works in other languages. Secondly, high-quality articles published recently may not have garnered adequate attention due to their brief publication period and lower citation counts.Ultimately, we only retrieved WOSCC, which may result in the omission of some studies.

## 5 Conclusion

In conclusion, China and the United States are at the forefront of global research on diabetic wounds, making significant contributions and fostering collaborations. The journal “Wound Repair and Regeneration” is a prominent publication in this field. Recent research has concentrated on various aspects of wound healing, including mechanisms, prognosis, treatment, and management. Current research hotspots emphasize therapeutic approaches such as antimicrobial methods, advanced wound care, extracellular vesicles, macrophage polarization, and injectable hydrogels. The growing recognition of prognostic analysis by scholars highlights the importance of integrating mechanistic and prognostic studies to enhance individualized patient evaluations. The more effective treatment strategies and intelligent clinical management techniques, offering more personalized and precise treatment for diabetic wound healing. These trends underscore the dynamic and evolving nature of research in diabetic wound therapy.

## Data Availability

The original contributions presented in the study are included in the article/supplementary material. Further inquiries can be directed to the corresponding author.
